# Marked increase of procalcitonin after the administration of anti-thymocyte globulin in patients before hematopoietic stem cell transplantation does not indicate sepsis: a prospective study

**DOI:** 10.1186/cc7749

**Published:** 2009-03-16

**Authors:** Helena Brodska, Tomas Drabek, Karin Malickova, Antonin Kazda, Antonin Vitek, Tomas Zima, Marketa Markova

**Affiliations:** 1 Institute of Clinical Biochemistry and Laboratory Diagnostics, General Teaching Hospital, U nemocnice 2, CZ-128 08 Prague 2, Czech Republic; 2 1st Faculty of Medicine, Charles University, Katerinska 32, CZ-121 08 Prague 2, Czech Republic; 3 Department of Anesthesiology, Safar Center for Resuscitation Research, University of Pittsburgh, 3434 Fifth Avenue, Pittsburgh, PA 15260, USA; 4 Institute of Hematology and Blood Transfusion, U nemocnice 1, CZ-128 20 Prague 2, Czech Republic

## Abstract

**Introduction:**

Procalcitonin (PCT) and C-reactive protein (CRP) are established markers of infection in the general population. In contrast, several studies reported falsely increased PCT levels in patients receiving T-cell antibodies. We evaluated the validity of these markers in patients scheduled for hemopoietic stem cell transplantation receiving anti-thymocyte globulin (ATG) during conditioning. We also assessed renal and liver functions and their relationship to PCT and CRP changes.

**Methods:**

Twenty-six patients without clinical signs of infection were prospectively studied. ATG was administered in up to three doses over the course of 5 days. PCT, CRP, white blood cell (WBC) count, urea, creatinine, glomerular filtration rate, bilirubin, alanin amino-transferase (ALT), and gamma-glutamyl transferase (GGT) were assessed daily during ATG administration. Pharyngeal, nose, and rectal swabs and urine samples were cultured twice weekly. Blood cultures were obtained if clinical symptoms of infection were present.

**Results:**

Baseline (BL) levels of both PCT and CRP before ATG administration were normal. WBC count decreased after ATG administration (*P *= 0.005). One day after ATG administration, both PCT and CRP levels increased significantly, returning to BL levels on day 4. Microbiological results were clinically unremarkable. There was no interrelationship between PCT levels and BL markers of renal or liver functions (*P *> 0.05 for all comparisons). Bilirubin and GGT were increased on days 2 to 5 and ALT was increased on day 3 (*P *< 0.05 versus BL). No difference in renal functions was observed. Three patients developed bacterial infection on days 7 to 11 with different dynamics of PCT and CRP. There was no association between the number of ATG doses and PCT levels or between the risk of developing infection and previous PCT levels.

**Conclusions:**

ATG triggered a marked early surge in PCT and CRP followed by a steady decrease over the course of 3 days. The dynamics of both PCT and CRP were similar and were not associated with infection. PCT levels were independent of renal and liver functions and were not predictive of further infectious complications. A direct effect of ATG on T lymphocytes could be the underlying mechanism. Hepatotoxic effect could be a contributing factor. Neither PCT nor CRP is a useful marker that can identify infection in patients receiving ATG.

## Introduction

Patients undergoing allogeneic hematopoietic stem cell transplantation are subjected to substantial immunoalteration that puts them at increased risk for acquiring infection. Immunosuppression during the conditioning phase before engrafting is induced by pharmacotherapy or total body irradiation. This results in significantly altered inflammatory response to infection. Clinical and laboratory markers of sepsis are of limited value. White blood cell (WBC) count is intentionally decreased and therefore has little informational value. Fever, another important clinical sign, can be caused by multiple factors or, by contrast, can be absent. Biochemical markers of inflammation – C-reactive protein (CRP) and procalcitonin (PCT) – were shown to be able to reliably diagnose infection in the general population. PCT seems to be superior in the early detection of inflammation. It also enables the differentiation between systemic inflammatory response syndrome (SIRS) and sepsis [1]. PCT concentrations are increased even in immunocompromised septic patients [2]. In neutropenic patients, PCT helps to identify those who require antibiotic treatment [3,4].

Anti-thymocyte globulin (ATG) is frequently used as part of a conditioning regimen in patients scheduled for allogeneic hematopoietic stem cell transplantation. In those patients, freedom from infection before engraftment is of the utmost importance. ATG administration could be associated with systemic reaction, including fever and hypotension, comparable to sepsis. The ATG-induced depletion of leukocytes makes one of the key diagnostic criteria of SIRS/sepsis [5] useless. Thus, biochemical markers of inflammation could be beneficial to differentiate between infectious versus non-infectious complications in this specific population. We prospectively evaluated the validity of CRP and PCT to diagnose infection in patients receiving ATG prior to hematopoietic stem cell transplantation. We also assessed renal and liver functions and their relationship to PCT and CRP changes.

## Materials and methods

In an observational non-randomized study, we prospectively evaluated a cohort of 26 adult patients indicated for an ATG conditioning regimen prior to hematopoietic stem cell transplantation. The patients were treated at the Institute of Hematology and Blood Transfusion in Prague, Czech Republic. The study was approved by the institutional review board. The purpose and procedures of the study were explained to participants, and written informed consent was obtained.

### Interventions

The conditioning regimen was selected according to the underlying disease. The ATG dose was selected according to donor-patient matching. A test dose of ATG (20 mg) was given after the baseline (BL) samples were obtained on day 0. Afterwards, ATG was administered once daily at a dose of 20 mg/kg during 6-hour infusion, and in those patients who were indicated for a total dose of 40 mg/kg, 20 mg/kg was administered the next day. Typically, there were two or three doses before transplantation.

Blood samples were drawn under aseptic conditions from a central venous catheter daily until transplantation. Heparinized plasma was used for PCT, CRP, and renal and liver function tests. PCT was measured by enzyme-linked fluorescence immunoabsorbent assay (VIDAS BRAHMS PCT; bioMérieux, Marcy l'Etoile, France). CRP was measured by turbidimetry (Modular SWA; Roche, Basel, Switzerland). WBC count was analyzed from K_3 _EDTA (ethylenediaminetetraacetic acid) samples by a blood count analyzer (Advia 120; Bayer, Leverkusen, Germany). Turbidimetry (Modular SWA) was also used for analyses of alanin amino-transferase (ALT), gamma-glutamyl transferase (GGT), and bilirubin to assess liver function and of urea, creatinine, and glomerular filtration rate (GFR) to assess renal function.

Pharyngeal, nose, and rectal swabs and urine samples were obtained prior to the initiation of treatment and twice weekly afterwards. Same samples plus blood cultures were obtained from all lumens of the central venous catheters and a peripheral vein when body temperature (measured in the axilla) increased above 37.5°C. Blood cultures were cultivated for both aerobic and anaerobic bacteria and for fungi (bacT/ALERT; bioMérieux).

### Statistical analysis

The plasma concentrations of biochemical markers are reported as mean ± standard deviation unless noted otherwise. Given a non-parametric distribution of results, concentrations of markers were compared using the Kruskal-Wallis test. Correlations between levels of markers were examined with the Spearman rank correlation coefficient. PCT levels were analyzed and categorized into quartiles according to a concentration to evaluate their association with the post-ATG febrile/infectious complications. All statistical analyses were performed using Statistica CZ 8.0 software (StatSoft Inc., Tulsa, OK, USA). All tests were two-tailed, and *P *values of less than 0.05 were considered statistically significant.

## Results

**Figure 1:**
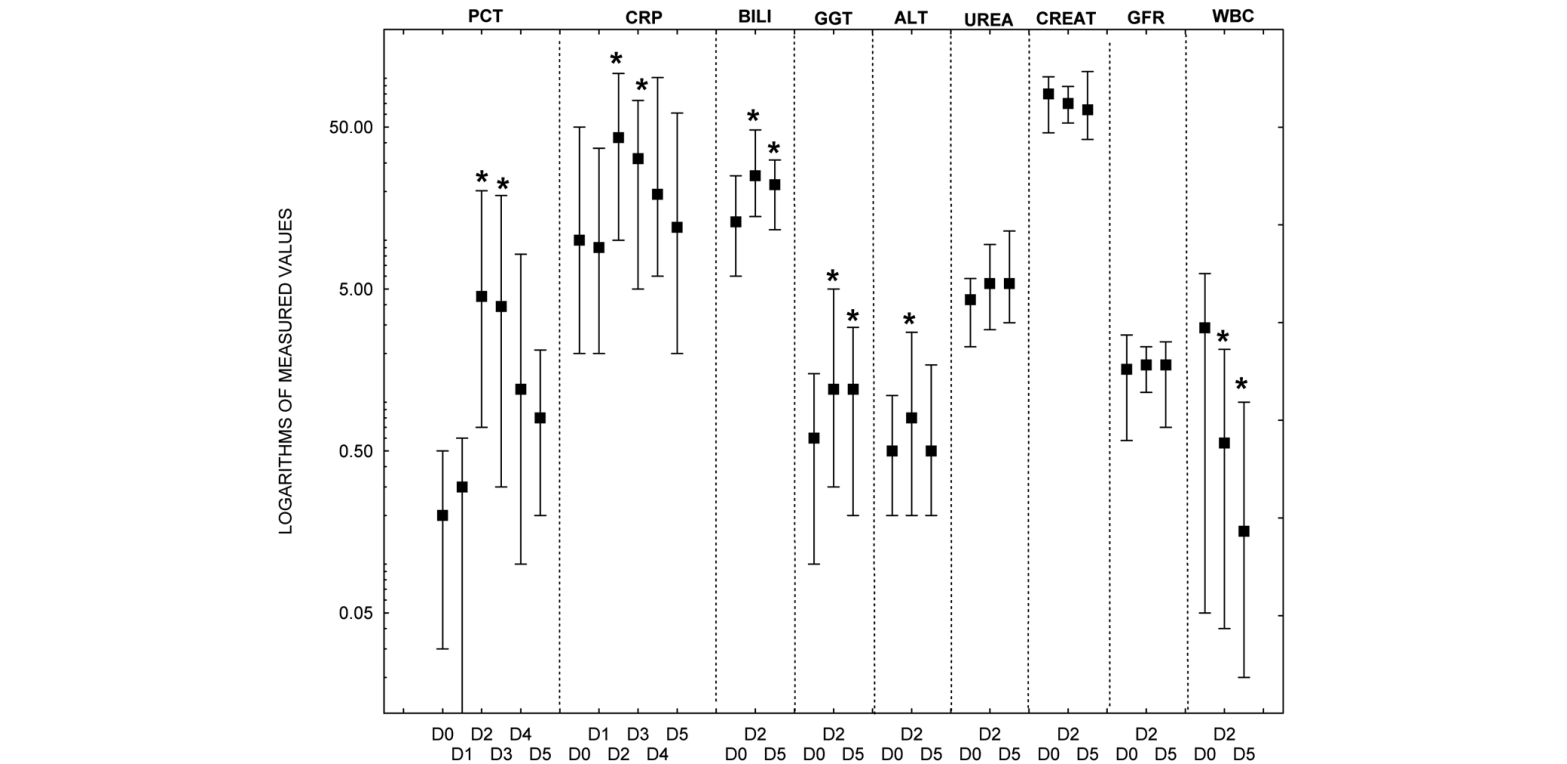
Dynamics of measured parameters during conditioning with anti-thymocyte globulin. Values are presented as mean ± standard deviation. *P < 0.05 versus baseline. ALT, alanin aminotransferase (normal: 0.1 to 0.78 μkat/L); BILI, bilirubin (normal: 2 to 17 μmol/L); CREAT, creatinine (normal: 44 to 104 μmol/L for females and 44 to 110 μmol/L for males); CRP, C-reactive protein (normal: <7 mg/L); Dx, day of conditioning regimen (see Results section for details); GFR, glomerular filtration rate (normal: 1.5 to 2.0 mL/s); GGT, gamma-glutamyl transferase (normal: 0.1 to 0.68 μkat/L); PCT, procalcitonin (normal: < 0.5 μg/L); urea (normal: 2.0 to 6.7 mmol/L for females and 2.8 to 8.0 mmol/L for males); WBC, white blood cell (count) (4.3 to 10.8 × 10^9^/L).

The demographic data and patients' characteristics are presented in Table 1. A significant increase in PCT was observed starting 24 hours after ATG administration. The initial surge was followed by a slow steady decrease. On day 5, PCT levels were still increased, but there was no statistical difference versus BL levels. The dynamics of CRP changes were similar to PCT, but CRP returned to BL values 1 day earlier. Similar statistically significant trends were observed for bilirubin and GGT. In contrast, ALT increased only transiently on day 2. There were no changes in urea, creatinine, or GFR during conditioning. Progressive depletion of leukocytes was observed over time (Figure 1). There was no statistically significant interrelationship of PCT levels and markers of renal or liver functions (*P *< 0.05 for all comparisons) (Table 2).

**Table 1:** Basic descriptive characteristics of patients

Characteristic	Number (percentage) of patients^a^
Gender	
Males	14 (54%)
Females	12 (46%)
Diagnosis	
Acute lymphoblastic leukemia	4 (17%)
Acute myeloid leukemia	7 (29%)
Myeloproliferative syndrome	5 (17%)
Chronic lymphatic leukemia	2 (8%)
Chronic myeloid leukemia	1 (4%)
Myelodysplastic syndrome	4 (13%)
Non-Hodgkin lymphoma	2 (8%)
Hodgkin disease	1 (4%)
Age at ATG treatment, years	
Mean (minimum, maximum)	43 (24, 62)
Number of ATG doses	
Two	11 (42%)
Three	12 (46%)
Four	3 (12%)
Post-ATG body temperature	
Normal body temperature	20 (77%)
Body temperature <37.5°C	5 (20%)
Body temperature >37.5°C	1 (3%)

^a^Values are presented as number (percentage) of patients, except for those of 'Age at ATG treatment', which are presented as mean (minimum, maximum). ATG, anti-thymocyte globulin.

**Table 2:** Interrelationship of procalcitonin and other measured laboratory parameters

	Procalcitonin					
*r*	Baseline	Day 1	Day 2	Day 3	Day 4	Day 5

Baseline values of						
CRP	0.04	0.17	0.06	0.29	0.32	0.03
BILI	0.04	0.35	0.23	0.09	0.21	0.13
GGT	0.03	0.22	0.07	0.22	0.19	0.02
ALT	0.10	0.14	0.17	0.07	0.21	0.07
GFR	0.12	0.32	0.08	0.04	0.09	0.16
Urea	0.11	0.22	0.31	0.25	0.43	0.19
CREAT	0.25	0.18	0.24	0.21	0.32	0.17

All measured significance levels are greater than 5% (n = 26, Spearman rank correlation test, *r*). Baseline = initial/pretreatment value. ALT, alanin aminotransferase; BILI, bilirubin; CREAT, creatinine; CRP, C-reactive protein; GFR, glomerular filtration rate; GGT, gamma-glutamyl transferase.

**Figure 2:**
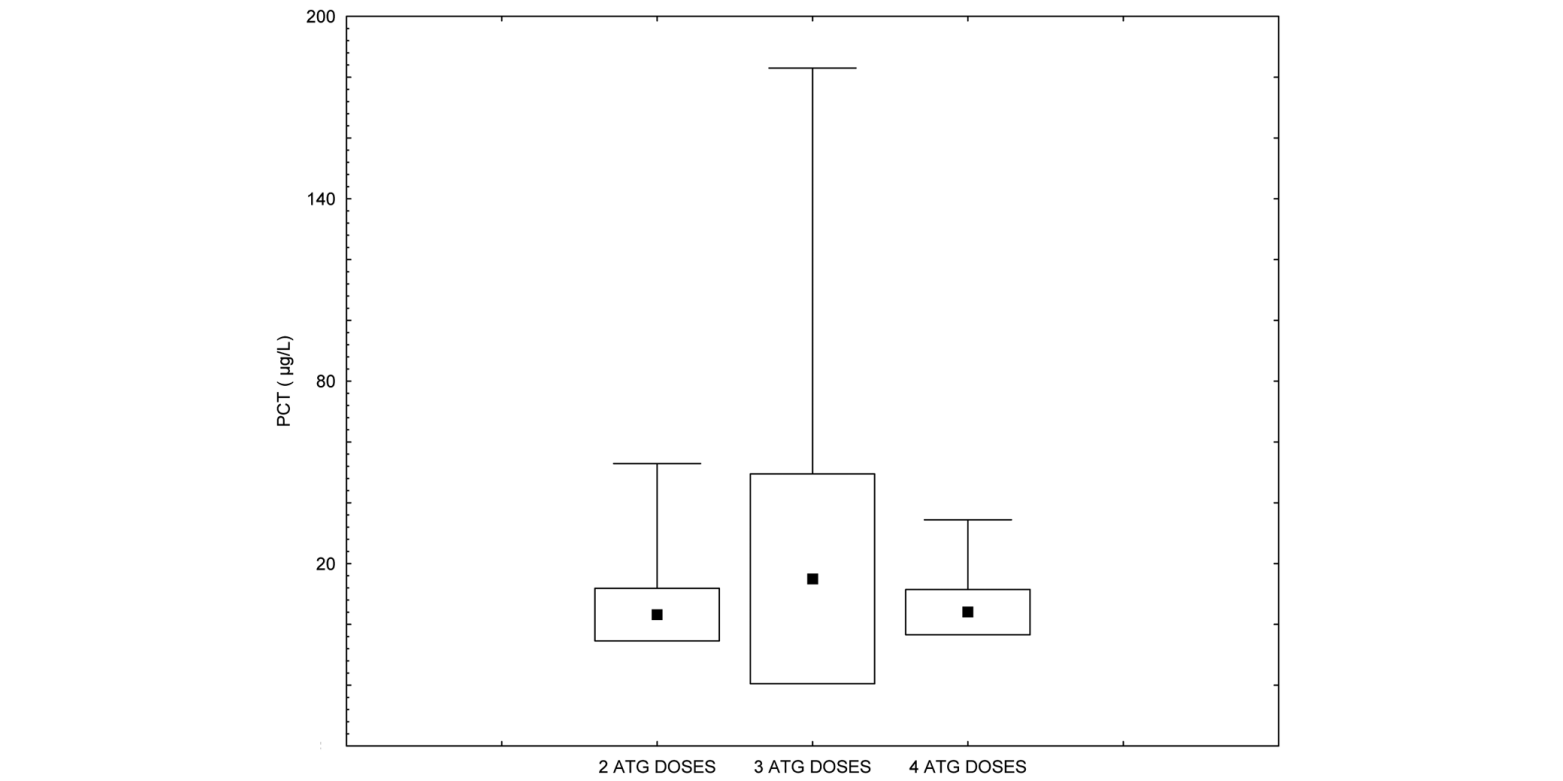
The relationship between the number of anti-thymocyte globulin (ATG) doses and procalcitonin (PCT) values. Black square markers represent the mean, boxes represent standard deviation, and whiskers represent the minimum/maximum for each group. *P *= 0.16 between groups.

The relative odds of post-ATG febrile complications did not increase significantly with each increasing quartile of BL PCT concentration. Thus, patients in the highest versus lowest quartile did not have any increase in risk. After adjustment for CRP, the concentration of PCT remained unassociated with the risk of post-ATG febrile complications (Table 3). There was no relationship between the number of ATG doses and PCT concentrations (*P *= 0.16) (Figure 2). Microbiological cultures of the pharyngeal, nose, and rectal swabs and urine samples yielded clinically insignificant results. Blood cultures obtained during conditioning did not grow any bacteria or fungi over the course of the 7-day inoculation period. Three patients developed sepsis 7 to 11 days after the ATG conditioning. The changes in PCT and CRP were different from those observed during conditioning (Table 4).

**Table 3:** Relative odds of post-anti-thymocyte globulin febrile complications according to procalcitonin concentration on transplantation day

	Quartile of procalcitonin concentration (range, μg/L)				
	
	Q1 (<1.6)	Q2 (1.6–4.1)	Q3 (4.1–14.9)	Q4 (>183)	*P *trend
Crude matched pairs					
OR	1.00	1.10	1.05	1.10	0.095
95% CI	-	0.03–0.85	0.25–5.87	2.51–59.87	
*P *value	-	0.26	0.11	0.09	
Adjusted for C-reactive protein					
OR	1.0	1.08	1.10	1.07	0.123
95% CI	-	0.026–0.99	0.31–5.75	2.41–58.35	
*P *value	-	0.34	0.10	0.09	

The relative odds of post-anti-thymocyte globulin (ATG) febrile/infectious complications did not increase significantly with each increasing quartile of baseline procalcitonin concentration, so patients in the highest versus lowest quartile did not have any increase in risk. After adjustment for C-reactive protein, the concentration of procalcitonin remained unassociated with the risk of post-ATG febrile complications. CI, confidence interval; OR, odds ratio.

**Table 4:** Procalcitonin and C-reactive protein concentrations and white blood cell count in patients with delayed sepsis

**Patient 1**	**D11**	**D12**	**D13**	**D14**	**D15**
PCT, μg/L	1.7	13.4	58	43	36
CRP, mg/L	124	150	173	295	300
WBC × 10^9^/L	0.12	0.23	0.35	0.50	0.48
**Patient 2**	**D7**	**D8**	**D9**	**D10**	**D11**
PCT, μg/L	7	8.1	5.3	3.1	1.1
CRP, mg/L	304	311	201	182	110
WBC × 10^9^/L	0.03	0.03	0.03	-	0.02

**Patient 3**	**D8**	**D9**	**D10**	**D11**	**D12**
PCT, μg/L	8.3	5.4	3.2	1.8	1.1
CRP, mg/L	270	330	370	260	180
WBC × 10^9^/L	0.18	0.22	0.15	0.12	0.10

The dynamics of markers of procalcitonin (PCT) and C-reactive protein (CRP) in patients who developed delayed sepsis on days 7 to 11 after conditioning with anti-thymocyte globulin (ATG) were different from the dynamics of increase in PCT and CRP that were observed during ATG conditioning. Dx, days after conditioning; WBC, white blood cell.

## Discussion

Both PCT and CRP have been shown to successfully diagnose systemic inflammation in various patient populations. A recent review of the role of PCT in febrile neutropenic patients suggested the superior role of PCT over other markers of infection in this population. Patients undergoing conditioning before hematopoietic stem cell transplantation represent a distinct population with significantly altered immune response. This creates a challenging scenario for clinical diagnosis of incipient infection which could be potentially catastrophic if not discovered early. Conditioning with ATG, a heterogeneous protein, can be associated with adverse reactions, mainly circulatory instability and/or respiratory insufficiency. The severity of this reaction could be highly individual and in selected cases could closely resemble sepsis. Ancillary biochemical tests that would readily detect infection would be of great benefit.

In our cohort of patients undergoing conditioning with ATG, we observed a characteristic early surge in PCT and CRP, followed by a steady decline to a near-normalization on day 4. Yet this was not associated with clinical infection, as monitored by microbiological cultures. Thus, neither PCT nor CRP proved useful as a valid complementary diagnostic tool in this setting.

Our observation has some support in the literature. Several previous reports suggested limited diagnostic value of PCT and CRP in the presence of anti-T-lymphocyte antibodies. In kidney transplant patients receiving pan-T-cell antibodies, Sabat and colleagues [6] found increased PCT concentrations that were comparable to those observed in sepsis. Similarly to our results, the early surge was observed 24 hours after the initiation of treatment. The increase in tumor necrosis factor-alpha (TNF-α) preceded the increase in PCT and was detectable as early as a few minutes after ATG administration. However, those patients did not have any infection [6]. Similarly, Zazula and colleagues [7] found increased PCT levels in orthotopic liver transplant recipients on the first day after surgery, with a more marked increase in those who received ATG. PCT decreased independently of further ATG administration in both groups of patients. No evidence of infection was present in either group [7].

Dornbusch and colleagues [8], in a small retrospective study, evaluated the diagnostic value of PCT and CRP in differentiating sepsis and febrile reaction after administration of anti-T-lymphocyte antibodies in pediatric patients. Neither PCT peak levels nor PCT concentrations 3 days after the onset of febrile reactions differed between septic patients and those receiving T-cell antibodies. Both PCT and CRP showed a trend similar to our observation, except for 5 (out of 21) patients whose CRP remained increased for 20 days irrespective of the duration of T-cell antibody administration [8].

Pihusch and colleagues [9] prospectively studied PCT, CRP, and interleukin-6 (IL-6) in 350 stem cell recipients. Conditioning with ATG increased all monitored markers. In neutropenic patients receiving ATG, there was no difference in PCT levels between patients with or without infections. After engraftment, PCT levels in patients without infections were significantly lower than in patients with infectious complications. However, the initial increase was less pronounced than in our cohort but persisted longer [9].

ATG is a mixture of antibodies against T cells with direct effect. The binding location of ATG on lymphocytes is mainly CD2, CD3, CD4/CD28, CD7^+^, LFA-1^+^, and ICAM-1, receptors characteristic for T cells. The main mechanism of action is opsonization and lysis by complement activation, leading to T-cell depletion.

The evidence suggests that the increase in PCT and CRP is not restricted to ATG. Similar reactions were observed after treatment with other T-cell antibodies, namely OKT-3. Treatment with monoclonal CD52 antibody alemtuzumab triggered even higher levels of PCT and CRP, comparable to Gram-negative sepsis [10].

The exact function, mechanism, and site of PCT production have yet to be fully unveiled. PCT activity has been identified in human leukocytes [11]. Others have suggested that liver [12,13], lungs, neuroendocrine cells, or various other tissues are possible sites of production [14,15].

A profound stimulatory effect of TNF-α on PCT mRNA levels [11] or PCT itself was observed. After TNF-α administration, PCT reached half-maximal concentrations within 8 to 12 hours earlier than CRP. It was suggested that PCT and acute-phase proteins such as CRP are induced by similar pathways [12]. It could be hypothesized that the T-cell antibody-induced increase of PCT is mediated via release of TNF-α and does not represent a direct effect of the antibody [16].

In our study, induction of PCT increase by ATG administration can be explained mainly by lymphocyte destruction. However, the contribution of hepatotoxicity must also be considered. As the dynamics of PCT and CRP changes are similar, we do not believe that a difference in half-time of these parameters plays a role here [17].

Bacterial infection with positive blood cultures developed in three patients in our study on days 7 to 11 after the last ATG dose. The dynamics of PCT and CRP changes in those patients during this episode were different from the characteristic course of ATG-induced changes and were not associated with actual WBC count.

Understanding the mechanisms of PCT release could help to elucidate its increase in other non-infectious conditions, as described below. The extent of this increase in our cohort was not dependent on initial liver and renal functions or WBC count. It was not predictive within the context of subsequent infectious complications and/or mortality. It could be speculated that the extent of PCT increase is related to a certain immunological body reserve that may not be fully reflected by actual WBC count or, more specifically, by transition of monocytes into macrophages.

A variety of clinical conditions, including cardiac surgery [18] and heatstroke [19], with increased PCT of non-infectious causes were reported. In healthy term neonates, a transient increase in PCT peaking at 24 hours after birth and gradually decreasing over the first 48 hours of life was observed [20]. In chronic renal insufficiency patients, results are conflicting. In pediatric patients, a small increase in BL PCT levels was observed [21]. Adult patients undergoing chronic hemodialysis treatment had normal PCT levels. In contrast, CRP was markedly increased in patients undergoing short- and long-term hemodialysis. PCT, but not CRP, was increased in patients on peritoneal dialysis [22]. An isolated increase in PCT, but not CRP or other inflammatory parameters, was registered in patients with medullar thyroid carcinoma [23,24].

Our study has limitations. We did not assess other markers (for example, IL-6, IL-8, TNF-α, endotoxin, serum amyloid A, or neopterin) that could be considered to augment the diagnosis of infection in neutropenic patients, as explored by others [25]. Also, we did not study any patients who would acquire infection during ATG conditioning and would represent a control group. This situation is extremely rare and only a few cases have been reported [9]. Based on our results and the available literature, we are not able to recommend any single test that would be able to rule in an infection in this specific patient population. A combination of a detailed clinical assessment and careful interpretation of collateral biochemical and microbiological tests probably remains to be the optimal approach targeted to individual patients. The mechanism of inflammatory reaction in neutropenic patients and prompt detection of infection in those patients need to be explored in future studies.

## Conclusions

ATG administration was associated with a characteristic rapid surge in both PCT and CRP followed by a steady decline over the next 3 days. This increase was not associated with systemic infection. The number of ATG doses was not related to the peak PCT concentrations. ATG induced an increase in liver function tests but not in markers of renal function. PCT levels were not altered by renal and liver functions or WBC count before conditioning. PCT seems to have no predictive value of future infectious complications. Both PCT and CRP have limited value in the diagnosis of infection during the administration of ATG.

## Key messages

• Anti-thymocyte globulin (ATG) administered during conditioning before hematopoietic stem cell transplantation triggered a marked increase in both procalcitonin (PCT) and C-reactive protein (CRP) with a peak at 24 hours after administration, followed by a steady decline over the next 3 days. This increase was not associated with systemic infection.

• The number of ATG doses was not related to the peak PCT concentrations.

• ATG induced an increase in liver function tests but not in markers of renal function. PCT levels were not altered by renal and liver functions or white blood cell count before conditioning.

• PCT seems to have no predictive value of future infectious complications.

• Both PCT and CRP have limited value in the diagnosis of infection during administration of ATG.

## Abbreviations

ALT: alanin amino-transferase; ATG: anti-thymocyte globulin; BL: baseline; CRP: C-reactive protein; GFR: glomerular filtration rate; GGT: gamma-glutamyl transferase; IL: interleukin; PCT: procalcitonin; SIRS: systemic inflammatory response syndrome; TNF-α: tumor necrosis factor-alpha; WBC: white blood cell.

## Competing interests

The authors declare that they have no competing interests.

## Authors' contributions

HB carried out the laboratory work and drafted the manuscript. MM was responsible for the patient care, ATG administration, sample timing, and collection and contributed to the preparation of the manuscript. KM performed the statistical analysis. AV participated in the transplantation and contributed to the study design. AK and TZ participated in the study design and helped to draft the manuscript. TD intellectually contributed to the preparation of the manuscript. All authors read and approved the final manuscript.
